# Genetic Polymorphism of Y-Chromosome in Turkmen Population from Turkmenistan

**DOI:** 10.3390/genes15121501

**Published:** 2024-11-22

**Authors:** Maxat Zhabagin, Assel Tashkarayeva, Alizhan Bukayev, Aigul Zhunussova, Georgy Ponomarev, Saltanat Tayshanova, Albina Maxutova, Dmitry Adamov, Elena Balanovska, Zhaxylyk Sabitov

**Affiliations:** 1 National Center for Biotechnology, Astana 010000, Kazakhstan; 2 DNK Shejire LLP, Astana 010000, Kazakhstan; 3 Research Institute for Jochi Ulus Studies, Astana 010000, Kazakhstan; 4 Astana International University, Astana 010000, Kazakhstan; 5 Research Centre for Medical Genetics, Moscow 115522, Russia; 6 Kazak Historical Society, Astana 010000, Kazakhstan; 7 Kh. Dosmukhamedov Atyrau University, Atyrau 060000, Kazakhstan; 8 L.N. Gumilyov Eurasian National University, Astana 010000, Kazakhstan

**Keywords:** population genetics, Y-chromosome, haplotype, haplogorup, network analysis, Turkmen population, Jochi Ulus, Central Asia

## Abstract

This study investigates the Y-chromosome genetic diversity of the Turkmen population in Turkmenistan, analyzing 23 Y-STR loci for the first time in a sample of 100 individuals. Combined with comparative data from Turkmen populations in Afghanistan, Iran, Iraq, Russia, and Uzbekistan, this analysis offers insights into the genetic structure and relationships among Turkmen populations across regions across Central Asia and the Near East. High haplotype diversity in the Turkmen of Turkmenistan is shaped by founder effects (lineage expansions) from distinct haplogroups, with haplogroups Q and R1a predominating. Subhaplogroups Q1a and Q1b identified in Turkmenistan trace back to ancient Y-chromosome lineages from the Bronze Age. Comparative analyses, including genetic distance (RST), median-joining network, and multidimensional scaling (MDS), highlight the genetic proximity of the Turkmen in Turkmenistan to those in Afghanistan and Iran, while Iraqi Turkmen display unique characteristics, aligning with Near Eastern populations. This study underscores the Central Asian genetic affinity across most Turkmen populations. It demonstrates the value of deep-sequencing Y-chromosome data in tracing the patrilineal history of Central Asia for future studies. These findings contribute to a more comprehensive understanding of Turkmen genetic ancestry and add new data to the ongoing study of Central Asian population genetics.

## 1. Introduction

The study of Y-chromosome genetic polymorphism holds significant relevance and demand in the exploration of paternal lineage genetic history, offering invaluable insights into the reconstruction of ancient migratory routes and the identification of common ancestors among diverse populations. These studies contribute substantially to the fields of population genetics, forensic science, and molecular anthropology [1]. To date, many populations around the world have been investigated using Y-chromosome markers [2]. However, among Central Asian populations, the Turkmens of Turkmenistan remain largely unexplored in this genetic context. Existing studies have predominantly focused on Turkmen populations in Afghanistan [3], Iran [4], Iraq [5], Russia [6], and Uzbekistan [7].

Turkmens are speakers of the Oghuz branch of the Turkic languages [8] with a global population exceeding 8.8 million. The majority of Turkmens reside in Turkmenistan, where they constitute the indigenous population, numbering approximately 5 million. Significant Turkmen communities also exist in Afghanistan (around 2.4 million), Iran (approximately 811,000), Uzbekistan (about 222,000), Pakistan (around 165,000), Turkey (approximately 125,000), Russia (around 41,000), and Tajikistan (about 20,000), according to data from the Joshua Project (https://joshuaproject.net/, accessed on 1 August 2024). The northern borders of modern Turkmenistan stretch from the steep escarpment of the Ustyurt Plateau to the Kara-Bogaz-Gol Bay in the northwest; from the Sarykamish Depression in the northeast to the Amu Darya River Valley in the east, where it runs along the right bank in the southern part, skirting the Sundukli sands from the north; and from the southwestern spurs of the Gissar Range in the far east to the Atrek River Valley and the Kopetdag Mountains in the south. The southern borders include the foothills of Paropamisus and the elevations of Badkhyz and Karabil, while in the west, Turkmenistan’s territory meets the Caspian Sea [9]. Covering a total area of 491,200 square kilometers, Turkmenistan is the second largest country in Central Asia by land area.

The formation of the Turkmen people involved contributions from diverse ancestral populations across various historical periods, including Sumerians, Hittites, Khwarezmians, Cimmerians, Scythians, Massagetae, Sarmatians, Parthians, Hyrcanians, Khurasanians, Alans, Huns, Hephthalites, Kipchaks, and Oghuz Turks [9]. Archeological investigations along the eastern Caspian Sea coast, including excavations at Jeitun Cave and the foothills of the Kopetdag Mountains, indicate that human settlement in present-day Turkmenistan began at least as early as the Neolithic. A prominent Neolithic culture in this region is the Jeitun Culture (6th–5th millennia BCE), recognized as one of the earliest sedentary agricultural societies in Central Asia. During the Bronze Age, Turkmenistan saw the emergence of the Bactria–Margiana Archaeological Complex (BMAC), a culturally significant center characterized by early proto-urban settlements like Namazga-Tepe, Altyn-Tepe, and Kara-Tepe. These settlements featured complex social structures and highly organized systems. In the Iron Age, ancient state formations, including Margiana, Bactria, Parthia, and Hyrcania, began to take shape within Turkmenistan. Between the fourth and sixth centuries, this region fell under the control of the Achaemenids, Alexander the Great, and the Parthian Empire. By the early eighth century, the Arab Caliphate had conquered the area, leading the local Turkic tribes to embrace Islam. As Arab influence waned, Turkmenistan came under the control of Oghuz tribes, which laid the foundations of the Turkmen ethnic identity. In the 12th century and in the beginning of the 13th century, the region was part of the Khwarezmian Empire and after 1220 came under rule of the Golden Horde until the early 16th century. Batu and Orda Ejen (sons of Jochi, grandsons of Genghis Khan) divided the Turkmen into two groups, Sainkhani (Turkmen tribes of Batu) and Essenkhani (Turkmen tribes of Orda Ejen) [10]. In subsequent centuries, Turkmen tribes spread along the eastern Caspian coast and the northwestern areas of Khwarezm. After the collapse of the Golden Horde in 1502 and the Uzbek conquests of Khorezm in the 1500–1510s, the Turkmens were part of the Khiva Khanate until the beginning of the 20th century. These complex historical processes contributed to the development of the Turkmen as a distinct ethno-cultural community that has preserved its traditions and cultural heritage across centuries despite extensive migrations throughout various regions of Asia.

The aim of this study is to investigate the Y-chromosome genetic diversity of the Turkmen population from Turkmenistan, analyzed here for the first time across 23 Y-STR loci. These data are examined within the context of other Turkmen populations from Afghanistan, Iran, Iraq, Russia, and Uzbekistan, providing a comprehensive comparative framework for understanding genetic relationships across these groups.

## 2. Materials and Methods

### 2.1. Sample and Data Collection

The study was conducted in accordance with the Declaration of Helsinki (1964) and was approved by the Institutional Review Board (or Ethics Committee) of the National Center for Biotechnology (protocol code No. 5, dated 16 October 2020). Unrelated healthy male volunteers of Turkmen descent from Turkmenistan (Dasoguz and Mary Province), whose ancestors had lived in the region for at least three generations, were recruited for the study. Recruitment was conducted in the city of Taraz, Kazakhstan, among students from Turkmenistan. Each volunteer signed an informed consent form and completed an ethnographic questionnaire, which included information about their tribal affiliation. Saliva samples were collected from 100 males of the Turkmen population using the Oragene DNA Self-Collection Kit (OG-500, DNA Genotek, Stittsville, ON, Canada).

A dataset was compiled for comparison, comprising five Turkmen populations (N = 379 samples), including Turkmen from Jawzjan Province, Afghanistan [3]; Turkmen from Northern Iraq [5]; Turkmen from the Republic of Karakalpakstan, Uzbekistan [7]; Turkmen from Golestan Province, Iran [4]; and Turkmen from the Stavropol region, Russia [6]. The Turkmen data were updated for 17 Y-STR markers and provided by Professor E.V. Balanovska.

To further contextualize these Turkmen populations, a comparison dataset of neighboring geographic populations was created, comprising 12 populations (N = 1415 samples) as follows: Tajiks and Uzbeks from Jawzjan Province, Afghanistan [3]; Arabs, Kurds, Syrians, and Yazidis from Northern Iraq [5]; Karakalpaks and Kazakhs from the Republic of Karakalpakstan, Uzbekistan, and Uzbeks from the Khorezm region, Uzbekistan [7,11]; Kazakhs from Western Kazakhstan [11]; and Iranians from Golestan and Razavi Khorasan Provinces, Iran [12].

### 2.2. DNA Isolation, Amplification, and STR Genotyping

DNA isolation from saliva samples was executed using the prepIT-L2P kit (DNA Genotek, Canada). Post-isolation, DNA concentrations were quantified with a Qubit 2.0 Fluorometer (Thermo Fisher Scientific, Waltham, MA, USA) using the Qubit dsDNA BR Assay Kit (Thermo Fisher Scientific, USA). DNA integrity and purity were evaluated via NanoDrop One spectrophotometry (Thermo Fisher Scientific, USA). PCR amplification was conducted using the PowerPlex Y23 System (Promega, Madison, WI, USA) on a SimpliAmp Thermal Cycler (Thermo Fisher Scientific, USA). An electrophoretic separation of PCR products was carried out using the WEN Internal Lane Standard 500 (Promega, USA) in Hi-Di Formamide (Thermo Fisher Scientific, USA) with an 8-capillary Applied Biosystems 3500 genetic analyzer equipped with POP-4 polymer and cathode and anode buffers (Thermo Fisher Scientific, USA). Control DNA 007 (Thermo Fisher Scientific, USA) served as the positive control, and ddH2O was employed as the negative control for each batch of Y-STR fragment analysis. The PowerPlex Y23 System (Promega, USA) included 17 standard Y-STR markers (DYS19, DYS385 a/b, DYS389I/II, DYS390, DYS391, DYS392, DYS393, DYS437, DYS438, DYS439, DYS448, DYS456, DYS458, DYS635, Y-GATA-H4) and 6 loci with high mutation rates (DYS481, DYS533, DYS549, DYS570, DYS576, DYS643). Samples displaying non-standard patterns, off-ladder alleles, or microvariant alleles were re-analyzed. Our laboratories have passed the YHRD Quality Control Test (YC000343) and contributed haplotype data accordingly. Adhering to the population genetic data guidelines [13], the haplotypes were submitted to the Y-Chromosome Haplotype Reference Database [2] (YHRD, http://www.yhrd.org, accessed on 1 August 2024) under accession number YA006030. The population genetic data are detailed in Supplementary Table S1.

### 2.3. Data Analysis

STR allele calls were analyzed from electropherograms using GeneMapper IDx v.1.6 software. Haplotype frequencies were determined through the Arlequin program version 3.5 [14]. The number of distinct haplotypes, the frequency of unique haplotypes, discrimination capacity, haplotype match probability, and haplotype diversity were calculated directly using Microsoft Office Excel. Haplotype diversity (HD) was computed using the formula HD = n*(1 − ∑pi^2)/(n − 1), where n is the sample size and pi is the frequency of the i-th haplotype [15]. Haplotype match probability (HMP) was calculated as the sum of the squared observed haplotype frequencies. Discrimination capacity (DC) was defined as the ratio of the number of distinct haplotypes to the total number of haplotypes. Forensic parameters, including the random match probability (RM), power of discrimination (PD), gene diversity (GD), polymorphism information content (PIC), power of exclusion (PE), typical paternity index (TPI), and the frequency for each locus, were calculated using STRAF 2.1.5 software [16]. This software also facilitated the illustration of Nei’s genetic distances [17] through dendrograms and multidimensional scaling (MDS). Pairwise genetic distances (RST) were computed using the “AMOVA and MDS” online tool on the Y-Chromosome Haplotype Reference Database website (http://www.yhrd.org, accessed on 1 August 2024). Median-joining networks were constructed using NETWORK v5.0.1.0 and NETWORK Publisher v2.1.2.5 [18] based on STR haplotype data without incorporating SNP contributions into the phylogeny. Intermediate alleles with repeat numbers were rounded to the nearest integer, and the DYS385a/b loci were excluded from network construction due to the inability to associate specific alleles with their respective copies. Haplotype affiliation to haplogroups was assessed using the Nevgen Y-DNA haplogroup predictor (https://www.nevgen.org/, accessed on 1 August 2024) and by comparing haplotypes from published data. High-resolution Y-chromosome haplogroup SNPs, their ages, and genetic connections with the closest modern samples from citizen science were determined using the Discovery phylogenetic tree from FamilyTreeDNA, as described in [19].

## 3. Results and Discussion

### 3.1. Analysis of Haplotype and Allele Diversity

**Figure 1:**
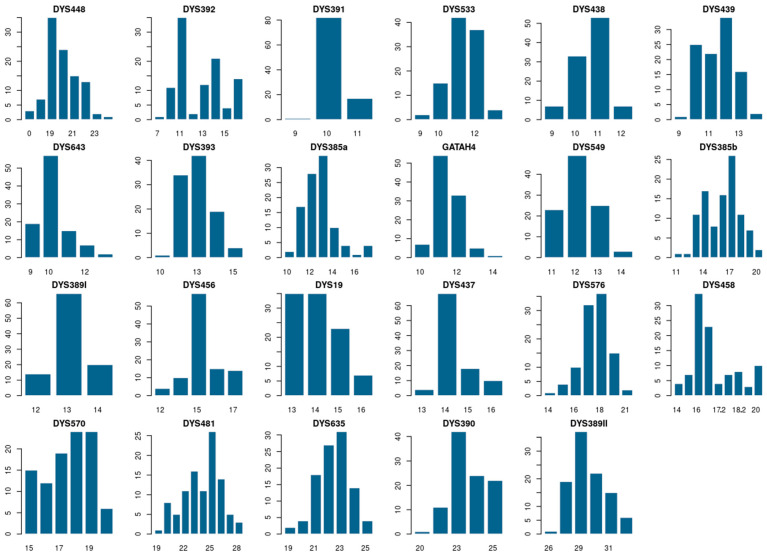
Distribution of allele frequencies for 23 Y-STRs in Turkmen population from Turkmenistan. Horizontal scales—allelic values of locus; vertical scale—allele occurrence.

The haplotype distribution of 23 Y-STR loci in a sample of 100 individuals from the Turkmen population of Turkmenistan is presented in Supplementary Table S1. Haplotype frequency analysis identified 83 distinct haplotypes, of which 71 are unique, as detailed in Supplementary Table S2. The haplotype match probability (HMP) was calculated to be 0.0029, and the haplotype diversity (HD) was found to be 0.995. The corresponding allelic frequencies varied from 0.01 to 0.82, as shown in Figure 1, with forensic parameters detailed in Supplementary Tables S3 and S4. The gene diversity (GD) ranged from 0.30 (DYS391) to 0.95 (DYS385a/b). All studied loci have GD values greater than 0.5, except for DYS391. Abnormal alleles are represented by microvariants for the DYS458 locus in 12 instances and deletions for the DYS448 locus in three instances, as listed in Supplementary Table S5. Microvariants for the DYS458 locus are characteristic of the J1 haplogroup, while deletions for the DYS448 locus are characteristic of the C2a1a1b1 haplogroup.

For comparison, the haplotype diversity and forensic parameters of 17 Y-STR loci from the Turkmen populations across Afghanistan, Iraq, Russia, Turkmenistan, and Uzbekistan are summarized in Table 1. The highest haplotype diversity is observed in the Turkmen populations of Iraq (0.996) and Turkmenistan (0.993), while the lowest is found in the Turkmen population of the Stavropol region of Russia (0.902).

### 3.2. Median-Joining Network and Haplogroup Analysis

**Figure 2:**
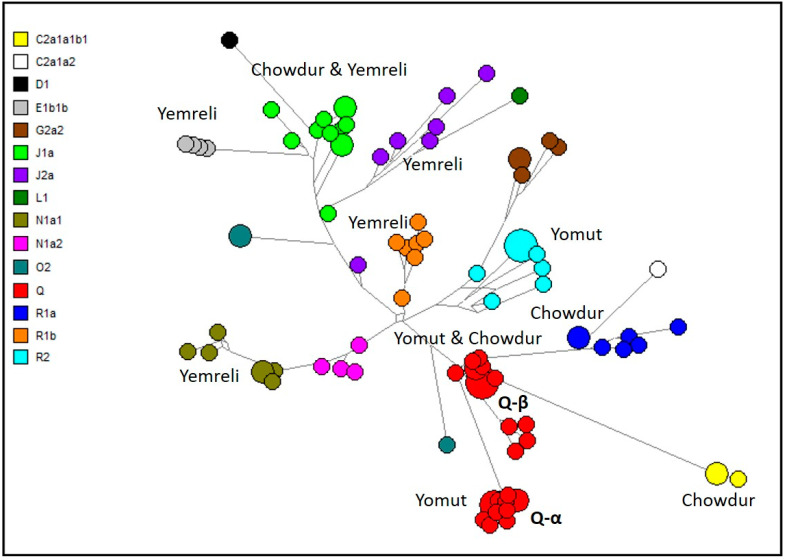
Median-joining network of 21 Y-STR haplotypes for Turkmen population from Turkmenistan distribution of predicted haplogroups. Circles represent haplotypes, with area proportional to sample size, and lines between them are proportional to number of mutational steps. Haplogroup categories represented in different colors are explained in top left legend.

Figure 2 illustrates a median-joining network of haplotypes for Turkmen populations from Turkmenistan, constructed using 21 Y-STR loci, with the DYS385a/b multilocus omitted. This Y-STR was omitted due to its multilocus nature, which can lead to ambiguity in allele assignment between the two loci and potentially inflate the number of inferred connections between haplotypes. This network emphasizes 15 principal Y-chromosome haplogroups, predicted via NevGen Genealogy Tools and detailed in Supplementary Table S1. The data reveal a high haplogroup diversity (HD  =  0.86), with Q (29%) and J1a (12%) as the most frequently observed haplogroups. Additional notable predicted core haplogroups include J2a, N1a1, R1a, and R1b, each constituting 7%, and R2 at 9% of the population. Other haplogroups, such as G2a2 (5%), E1b1b (4%), and N1a2 (4%), also contribute to the diversity within the sample. Lower-frequency haplogroups include C2a1a1b1 (3%), O2 (3%), C2a1a2 (1%), D1 (1%), and L1 (1%) (Figure 2).

The most represented tribes among the Turkmen sample from Turkmenistan are Chowdur (20%), Yemreli (21%), and Yomut (46%), while others (13%) comprise Ersari, Goklen, Sariq, and Teke. The network (Figure 2) highlights the predominant haplogroups associated with each Turkmen tribe. Haplogroup Q is major among Chowdur (45%) and Yomut (41%), whereas J1a is more prevalent in Yemreli (19%), alongside haplogroups E1b1b (14%), N1a1 (14%), R1b (14%), and J2a (10%).

**Figure 3:**
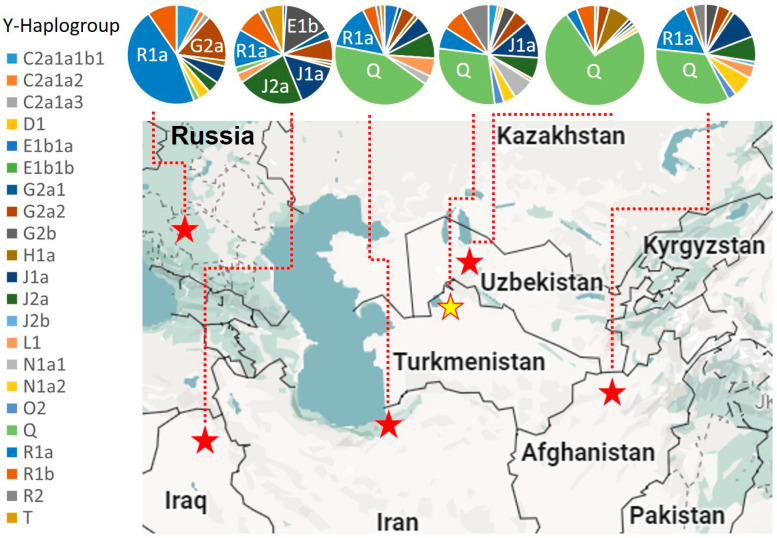
Y-haplogroup spectrum in Turkmen population from different geographical locations.

**Figure 4:**
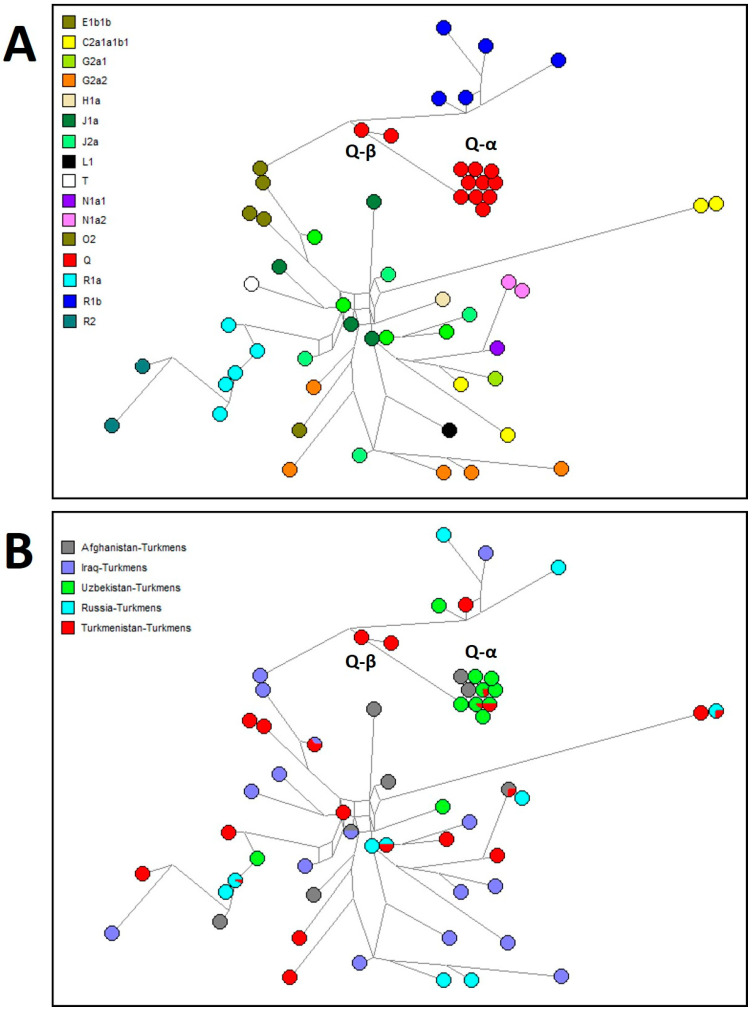
Median-joining network of 15 Y-STR haplotypes for Turkmen population. (**A**) Haplogroup affiliation. (**B**) Geographical affiliation.

**Figure 5:**
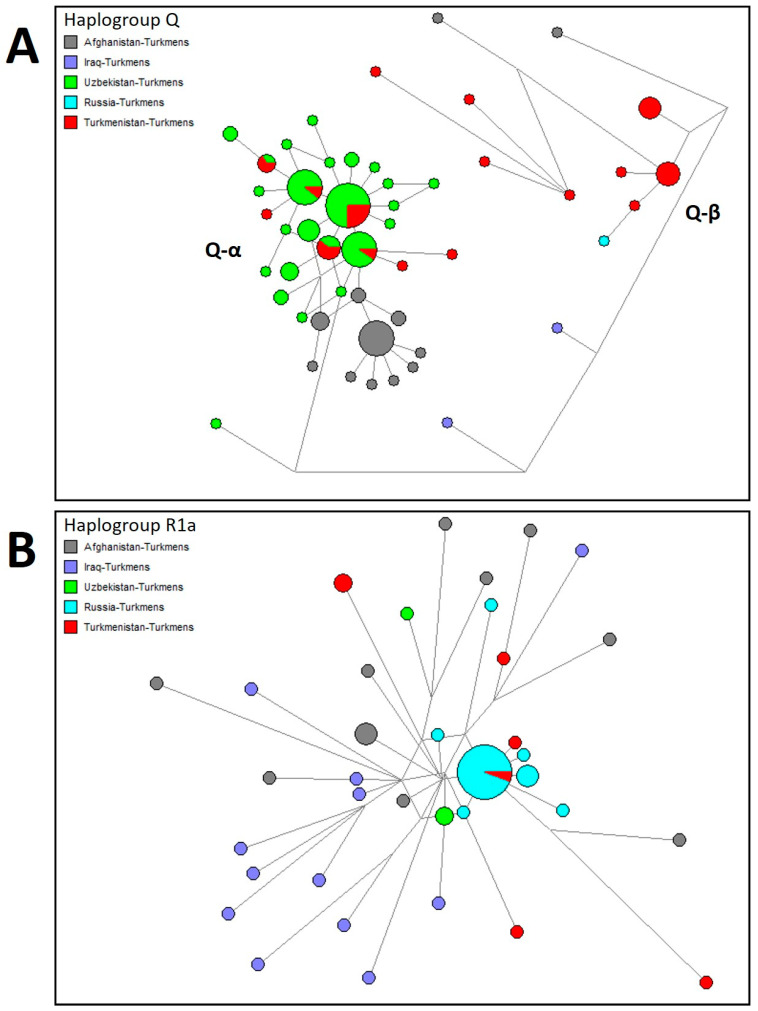
Median-joining network of 17 Y-STR haplotypes for Turkmen population belonging to (**A**) Q haplogroup and (**B**) R1a haplogroup. Circles represent haplotypes, with the area proportional to sample size, and lines between them proportional to the number of mutational steps.

For comparative analysis, the core haplogroup predictions for Turkmen populations across Afghanistan, Iran, Iraq, Russia, Turkmenistan, and Uzbekistan are presented in Figure 3 and Figure 4 and Supplementary Table S6. A median-joining network for all Turkmen based on common 15 Y-STR loci is shown in Figure 4. Approximately 74% of Y-chromosome variation among Turkmen is accounted for by six haplogroups with frequencies exceeding 5%, with Q at 29%, R1a at 14%, J1a at 9%, J2a at 9%, R1b at 7%, and G2a2 at 6%. A pronounced founder effect (lineage expansion) is observed only for haplogroups Q and R1a, as depicted in Figure 5, whereas no such effect is detected for other haplogroups in median networks (Supplementary Tables S1–S4).

Haplogroup Q encompasses no less than one-third of the Y-chromosome diversity in Turkmen populations from Turkmenistan (29%), Afghanistan (34%), Iran (42.6%), and Uzbekistan (73%), while it is rare among Turkmen in Iraq and Russia (2%). In Uzbekistan (particularly Karakalpakstan), the Turkmen population predominantly comprises the Yomut tribe (88%), for whom haplogroup Q (71%) is major [7], similar to the Yomut in Turkmenistan (46%). Within the median networks (Figure 5A), haplogroup Q is represented by two clusters, Q-α and Q-β. Samples from these clusters were previously sequenced using next-generation methods [20], identifying two subhaplogroups, Q1a-F1096 and Q1b-M346. Subhaplogroup Q1b is represented by lineage Q-Y148637, which primarily includes Turkmen samples from Turkmenistan (Figure 5A). According to FamilyTreeDNA’s Discovery phylogenetic tree, lineage Q-Y148637 diverged from the ancestral lineage Q-YP4024 around 550 BCE (https://discover.familytreedna.com/y-dna/Q-YP4024/classic, accessed on 1 August 2024), with its closest sibling lineage, Q-YP4055, predominantly found among samples from the Chechen Republic. Subhaplogroup Q1a is represented by lineage Q-Z35995, which diverged from ancestral lineage Q-YP1682 around 1100 CE (https://discover.familytreedna.com/y-dna/Q-YP1682/classic, accessed on 1 August 2024) and is found in Afghanistan, Turkmenistan, and Uzbekistan (Figure 5A). Both Q-Y148637 and Q-Z35995 share close affinities with samples from the Iron Age Central Asian cultural group [21]. The earliest known Q haplogroup sample (Gonur 6119) from present-day Turkmenistan was discovered at Gonur-Depe, a Bronze Age site within the Bactria–Margiana Archaeological Complex (BMAC) (2133-1946 BCE) [22].

The second haplogroup exhibiting a strong founder effect (lineage expansion) is R1a (Figure 5B), marked by high accumulation (46%) among Turkmen populations in Russia. The Russian Turkmen, descendants of migrants to the Stavropol region from Mangyshlak Peninsula during the late 17th to early 18th centuries, are primarily represented by the Soyunaji tribe (53%), where R1a reaches a frequency of 82%. In other Turkmen populations, R1a is moderately represented in Afghanistan (16%), Iraq (13%), and Iran (14.5%), while it is less prevalent in Turkmenistan (7%) and Uzbekistan (4%). Within haplogroup R1a, the Turkmen exhibit lineages R-PRX21 and R-FTC79947 [20], corresponding to the Asian and European subhaplogroups R1a1a1b2-Z93 and R1a1a1b1a-Z282, respectively [23]. The earliest R1a sample (Takhirbai 382) from present-day Turkmenistan was found at Takhirbai-Depe, a Bronze Age settlement in the BMAC (916-796 BCE) [24]. Supplementary Table S7 provides high-resolution Y-chromosome haplogroups for previously studied Turkmen [20] and identifies their genetic affinities with the closest modern samples from public genealogical projects (FamilyTreeDNA) and ancient samples from academic studies [25,26,27,28,29,30,31,32,33,34,35,36,37], integrated within FamilyTreeDNA’s Discovery phylogenetic tree.

### 3.3. Population Comparison Analysis

**Figure 6:**
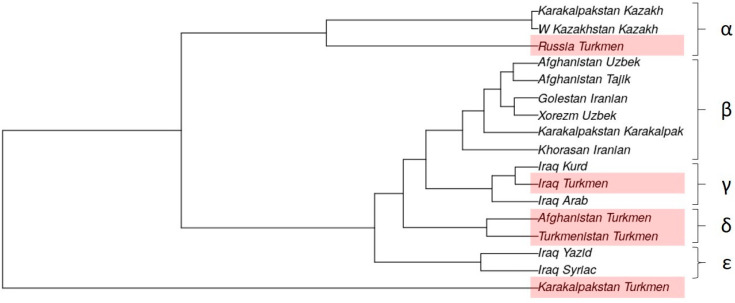
Phylogenetic relationship between Turkmen populations and geographical neighbor published populations based on Nei’s genetic distance (generated using 17 Y-STR analysis for forensics software (STRAF) version 2.1.5 [16]).

**Figure 7:**
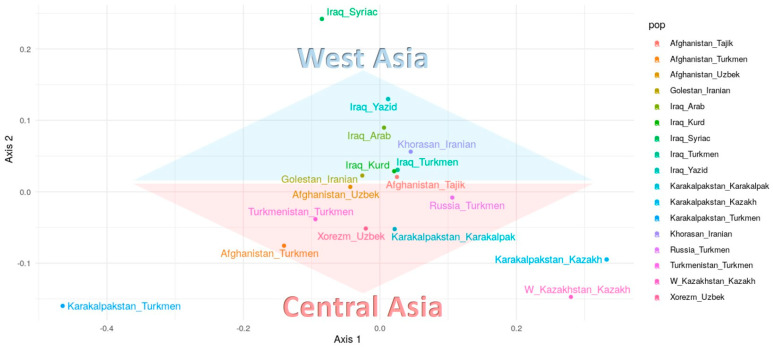
MDS based on Nei’s genetic distance between Turkmen populations and geographical neighbor published populations (generated using 17 Y-STR analysis for forensics software (STRAF) version 2.1.5 [16]).

The genetic position of the Turkmen population from Turkmenistan was determined in relation to other regional Turkmen populations and their neighboring populations based on 17 Y-STR loci. Sixteen populations were included in the analysis, namely Turkmen, Tajiks, and Uzbeks from Afghanistan [3]; Turkmen, Arabs, Kurds, Syrians, and Yazidis from Northern Iraq [5]; Turkmen, Karakalpaks, Kazakhs, and Uzbeks from Uzbekistan [7,11]; Kazakhs from Western Kazakhstan [11]; Iranians from the Golestan and Razavi Khorasan Provinces in Iran [12]; and Turkmen from the Stavropol region of Russia [6]. Pairwise genetic distances (RST) among these populations are presented in Supplementary Table S8. The genetic relationships among the populations were also visualized through a dendrogram (Figure 6) and multidimensional scaling (MDS) (Figure 7) based on Nei’s genetic distances calculated from the 17 Y-STR loci.

The genetic distance matrix reveals that the Turkmen from Turkmenistan are genetically closest to the Turkmen from Afghanistan (d = 0.0218), the Turkmen from Iraq are closest to the Iraqi Arabs (d = 0.0024), the Turkmen from Uzbekistan are closest to the Turkmen from Afghanistan (d = 0.1635), and the Turkmen from Russia are closest to the Afghan Tajiks (d = 0.0223). Genetic distinctiveness is observed in the Turkmen populations from Uzbekistan (Karakalpakstan) and Russia, which exhibit founder effects (lineage expansions) characterized by specific haplogroups, Q and R1a, respectively. The Turkmen from Uzbekistan (Karakalpakstan) show notable genetic distance from their closest geographic neighbors, who are the Karakalpaks (d = 0.3464), Uzbeks (d = 0.3391), and the Kazakhs (d = 0.5377).

The dendrogram (Figure 6) identifies five distinct clusters. One cluster, the δ-cluster, exclusively comprises Turkmen populations, bringing together the Turkmen from Turkmenistan and Afghanistan. Two clusters represent Iraqi populations; the γ-cluster includes the Turkmen, Arabs, and Kurds of Iraq, while the ε-cluster comprises the Yazidis and Syrians. The largest β-cluster does not contain any Turkmen groups. The α-cluster characterizes the Mangyshlak Peninsula, a region historically significant for the western Kazakh tribes and the origin of the Turkmen who migrated to the Stavropol region four centuries ago. The Turkmen of Uzbekistan (Karakalpakstan) do not form a cohesive cluster.

In the multidimensional scaling (MDS) analysis (Figure 7), two primary clusters are distinguishable, a Near Eastern cluster and a Central Asian cluster. With the exception of the Iraqi Turkmen, all Turkmen populations align with the Central Asian cluster.

## 4. Conclusions

This study presents the first data on Y-chromosome polymorphism among the Turkmen population of Turkmenistan based on 23 Y-STR loci. Additionally, it consolidates all available information on Y-chromosome variability among the Turkmen of Afghanistan, Iran, Iraq, Russia, and Uzbekistan in the context of neighboring geographic populations. The Turkmen of Turkmenistan exhibit high haplotypic diversity, shaped by founder effects (lineage expansions) of different haplogroups within distinct clans. Some of these clans constitute a significant portion of the Turkmen in Russia and Uzbekistan and due to founder effects (lineage expansions), these populations display pronounced genetic differentiation from their neighboring geographic populations. The most frequent haplogroups among the Turkmen with notable founder effects (lineage expansions) are Q and R1a. Both haplogroups have been present in this region since the Bronze Age. Among the Turkmen of Turkmenistan, subhaplogroups Q1a and Q1b are observed, whereas only Q1a is found in other Turkmen groups. Genetically, the Turkmen of Turkmenistan are most similar to the Turkmen of Afghanistan and Iran. Except for the Iraqi Turkmen, all Turkmen populations are characterized by a Central Asian genetic affinity and Y-chromosome variability spectrum. The acquisition of new deep-sequencing Y-chromosome data from the Turkmen offers an opportunity for a detailed study of Central Asian kinship to reconstruct the patrilineal genetic history of the region within the context of archeological, ethnographic, historical, and linguistic evidence.

## Figures and Tables

**Table 1:** Haplotype diversity and forensic parameters of 17 Y-STR haplotypes in the Turkmen populations.

Population	Number of Samples	Number of Distinct Haplotypes	Frequency of Unique Haplotypes	Discrimination Capacity	Haplotype Match Probability	Haplotype Diversity
Turkmenistan Turkmen	100	76	60%	76%	0.007	0.993
(this study)						
Russia Turkmen	53	29	42%	55%	0.098	0.902
(updated Skhalyakho et al., 2016) [6]						
Uzbekistan Turkmen	83	46	41%	55%	0.032	0.968
(Zhabagin et al., 2017) [7]						
Iraq Turkmen	102	86	73%	84%	0.004	0.996
(Dogan et al., 2017) [5]						
Afghanistan Turkmen	73	55	63%	75%	0.017	0.983
(Di Cristofaro et al., 2013) [3]						

## Data Availability

Data are available in a publicly accessible repository. The data presented in this study are openly available in the Y-Chromosome Haplotype Reference Database at [http://www.yhrd.org, accessed on 1 August 2024] [accession number YA006030].

## Supplementary Materials

The following supporting information can be downloaded at: https://www.mdpi.com/article/10.3390/genes15121501/s1. Table S1: The haplotype distributions of 23 Y-chromosomal STRs in the Turkmen population (N = 100); Table S2: The haplotype frequencies of 27 Y-chromosomal STRs in the Turkmen population (N = 100); Table S3: Allele frequencies and forensic parameter values for 21 single-locus Y-STRs in the Turkmen population (N = 100); Table S4: Locus-specific haplotypes frequencies and forensic parameter values for DYS385a/b in the Turkmen population (N = 100); Table S5: Abnormal alleles detected in the Turkmen population; Table S6: The haplotype distributions of 17 Y-chromosomal STRs in the Turkmen population from Afghanistan, Iraq, Russia, and Uzbekistan; Table S7: The high-resolution haplogroup of Y-chromosome in the Turkmen population; modern and ancient connection from Discovery by FamilyTreeDNA; Table S8: Pairwise genetic distance (RST) between Turkmen and neighboring populations on 17 Y-STRs.
